# Infection of Pro- and Anti-Inflammatory Macrophages by Wild Type and Vaccine Strains of Measles Virus: NLRP3 Inflammasome Activation Independent of Virus Production

**DOI:** 10.3390/v15020260

**Published:** 2023-01-17

**Authors:** San Suwanmanee, Shristi Ghimire, Jerome Edwards, Diane E. Griffin

**Affiliations:** W. Harry Feinstone Department of Molecular Microbiology and Immunology, Johns Hopkins Bloomberg School of Public Health, 615 N. Wolfe Street, Baltimore, MD 21205, USA

**Keywords:** measles virus, vaccine attenuation, macrophages, inflammasome, IL-1β, IL-6, IL-18, TNFα

## Abstract

In humans and non-human primates, wild type (WT) measles virus (MeV) replicates extensively in lymphoid tissue and induces an innate response characteristic of NF-κB and inflammasome activation without type I interferon. In contrast, the live attenuated MeV vaccine (LAMV) replicates poorly in lymphoid tissue with little detectable in vivo cytokine production. To characterize the innate responses of macrophages to WT MeV and LAMV infection, we analyzed primary human monocyte-derived macrophages and phorbol myristic acid-matured monocytic THP-1 cells (M0) polarized to inflammatory (M1) and anti-inflammatory (M2) phenotypes 24 h after MeV infection. LAMV infected macrophages more efficiently than WT MeV but produced less virus than WT MeV-infected macrophages. Both strains induced production of NF-κB-responsive cytokines IL-6 and TNFα and inflammasome products IL-1β and IL-18 without evidence of pyroptosis. Analysis of THP-1 cells deficient in inflammasome sensors NOD-like receptor pyrin (NLRP)3, IFN-γ-inducible protein 16 (IFI16) or absent in melanoma (AIM)2; adaptor apoptosis-associated speck-like protein containing a CARD (ASC) or effector caspase 1 showed that IL-18 production was dependent on NLRP3, ASC, and caspase 1. However, M1 cells produced IL-1β in the absence of ASC or caspase 1 indicating alternate pathways for MeV-induced pro-IL-1β processing. Therefore, the innate response to in vitro infection of macrophages with both LAMV and WT MeV includes production of IL-6 and TNFα and activation of the NLRP3 inflammasome to release IL-1β and IL-18. LAMV attenuation impairs production of infectious virus but does not reduce ability to infect macrophages or innate responses to infection.

## 1. Introduction

Measles is a highly contagious rash disease caused by measles virus (MeV), an enveloped, non-segmented, negative sense RNA virus. Despite availability of a safe and efficacious live attenuated measles vaccine (LAMV) measles cases and deaths have been increasing [1]. Infection with wild type (WT) MeV has profound effects on the immune system with decreased antibody diversity and increased susceptibility to other infections along with long term persistence of MeV RNA in lymphoid tissue and induction of lifelong immunity [2,3,4,5,6,7]. Although the adaptive immune response that is manifested by T cell infiltration into sites of virus replication and appearance of the maculopapular rash have been well characterized, infection is followed by a long clinically silent incubation period and innate responses to MeV infection are poorly understood.

Innate immune responses that differentiate self from non-self and prompt defensive responses to pathogens are essential for host survival. Pathogen-associated molecular patterns (PAMPs) recognized by host pattern recognition receptors (PRRs) are key components of this process and the signaling induced often promotes early control of pathogen replication, inflammation, and development of the adaptive immune response [8]. The most common viral PAMPS are nucleic acids with features or cellular locations distinct from host RNA or DNA. PRRs include RNA helicases and other specialized RNA- and DNA-binding proteins that initiate activation of signaling pathways that lead to phosphorylation of transcription factors interferon (IFN) regulatory factor 3 and NF-κB to produce IFN and inflammatory cytokines such as interleukin (IL)-6 and chemokines such as IL-8/CXCL8. A third pathway involves induction, assembly, and activation of inflammasomes for production of cytokines IL-1β and IL-18. 

Most viruses that cause disease in humans are sensitive to the antiviral effects of IFN and IFN production is commonly detected during the febrile response to infection. However, MeV encodes proteins that effectively inhibit the induction of and response to type I and III IFNs [9,10]. This allows MeV to replicate and spread systemically for several days before the disease-associated fever and rash of the adaptive immune response appear, virus replication is controlled, and virus clearance begins. However, innate responses to MeV are not absent. Transcriptomic analysis of peripheral blood mononuclear cells (PBMCs) from children with measles shows upregulation of mRNAs for IL-1β, CIAS-1/NLRP3, tumor necrosis factor (TNF)α, CCL4/MIP-1β, CXCL2/GRO-β, CXCL8, and TNFAIP3/A20 consistent with NF-κB signaling and inflammasome priming [11]. Furthermore, natural infection in humans and experimental WT MeV infection of rhesus macaques are associated with increases in plasma levels of IL-6, CXCL8, TNFα, IL-1β, and IL-18 proteins suggesting that the primary innate responses consist of NF-κB signaling and inflammasome activation without induction of type I IFN [11,12,13,14,15]. 

However, MeV is likely to be important for induction of innate responses and dependent on virus strain because both systemic spread of virus and cytokine and chemokine production are limited after infection with LAMV compared to WT MeV [13,15]. Restricted LAMV spread is associated with limited LAMV replication in lymphoid tissue and restricted in vitro virus production by primary lymphoid and myeloid cells 24 h after LAMV infection compared to WT MeV [15,16]. Myeloid cells, including tissue macrophages that can have both pro-inflammatory (M1) and anti-inflammatory (M2) phenotypes [17,18] are one of the target cells for MeV infection [19,20,21,22,23] and an important site for induction of innate responses to infection including inflammasome activation. 

Inflammasomes are multicomponent complexes composed of sensors, downstream effectors and in some cases adapters to link the two [24]. Sensors that lead to inflammasome activation in the cytosol belong to several families of proteins including the nucleotide-binding oligomerization domain (NOD)-leucine-rich repeat domain receptor (NLR) and PYHIN families. NLR family proteins have four types of N-terminal domains: acidic transactivation (NLRA), pyrin (NLRP), caspase recruitment (CARD; NLRC), and baculoviral inhibitory repeat (BIR; NLRB). PYHIN family proteins have C-terminal HIN and N-terminal pyrin domains and include absent in melanoma 2 (AIM2) and IFN-γ-inducible protein 16 (IFI16) [24]. Effectors are caspases activated to cleave pro-IL-1β and pro-IL-18 for release of mature IL-1β and IL-18. Apoptosis-associated speck-like protein containing a CARD (ASC) is an adaptor. Expression of inflammasome components is cell-type specific with myeloid and epithelial cells serving as important sites during viral infections [25,26]. 

The process of inflammasome activation is most clearly described for bacteria, but viruses also activate the inflammasome cascade in infected cells with important consequences for disease pathogenesis [27,28,29,30,31,32]. Inflammasome activation requires two steps. During the first priming step PAMP induction of PRR-mediated NF-κB signaling increases the expression of inflammasome components such as NLRP3 and pro-IL-1β that have limited expression in resting macrophages [33]. The second step triggers the oligomerization and assembly of the inflammasome complex for caspase 1-mediated processing of cytokine precursors and is typically induced by a change in the intracellular ion concentration, mitochondrial function, or reactive oxygen species [34]. Assembly of these supramolecular structures in the cytoplasm of infected cells with activation of caspase-1 can drive subsequent inflammation with production of IL-1β and IL-18 as well as cell death by pyroptosis [24,34].

To better understand the differential induction of cytokines in response to infection with WT MeV and LAMV, we have analyzed replication and induction of innate responses for differentiated primary human macrophages and phorbol 12-myristate 13-acetate (PMA)-matured and differentiated human THP-1 monocytic cells with specific inflammasome component deficiencies. We demonstrate that WT MeV and LAMV infect macrophages similarly, but that cells infected by WT virus produce infectious virus within 24 h while cells infected with LAMV do not. Despite differences in virus production, both strains of MeV induce inflammasome activation with both IL-1β and IL-18 production by pro (M1) and anti-inflammatory (M2) THP-1 cells with little evidence of pyroptosis. Production of IL-18 was dependent on NLRP3, ASC and caspase 1 while IL-1β production was dependent on NLRP3 but occurred in the absence of ASC or caspase 1 in M1-differentiated cells. 

## 2. Materials and Methods

### 2.1. Cells

Parental THP-1 cells and THP-1 cells with CRISPR-induced deficiencies in NLRP3, IFI16, ASC, AIM2, or caspase-1 were obtained from Victor DeFilippis (Oregon Health and Science University) [35] and provided to us by Andrea Cox (Johns Hopkins University School of Medicine) with permission. Cells were maintained in RPMI 1640 (Invitrogen, Waltham, MA, USA) supplemented with 10% heat-inactivated fetal bovine serum (Atlanta Biologicals, Flowery Branch, GA, USA), 1% sodium-pyruvate, 1% L-glutamine, 1% MEM non-essential amino acids, and 1% penicillin-streptomycin. For differentiation into macrophages (M0), cells were stimulated overnight with PMA (100 ng/mL) in RPMI 1640, 2% FBS, 1% L-glutamine, 1% penicillin-streptomycin [28]. For pro-inflammatory macrophage (M1) differentiation, M0 macrophages were further cultured with LPS (100 ng/mL; Cell Signaling, Danvers, MA, USA) and recombinant human IFN-γ (20 ng/mL, Stemcell Technologies, Vancouver, BC, Canada) for 24 h. For anti-inflammatory macrophage (M2) differentiation, M0 cells were cultured with 20 ng/mL human recombinant IL-4 (Stemcell Technologies) for 24 h [36,37]. 

Primary human monocytes were isolated from PBMCs separated from normal human donor buffy coat cells (American Red Cross) on Ficoll-Paque Premium (Cytiva, Marlborough, MA, USA) and isolated using the immunomagnetic negative selection EasySepHuman Monocyte Isolation kit. Monocytes were maintained in ImmunoCult-SF Macrophage medium (Stemcell Technologies) and cultured with 50 ng/mL human recombinant macrophage colony stimulating factor (M-CSF; Stemcell Technologies) for a total of 8 days. At day 6, 10 ng/ mL LPS and 50 ng/ mL IFN-γ were added for M1 differentiation and 10 ng/ mL IL-4 was added for M2 differentiation. 

### 2.2. Viruses, Virus Infection and Inflammasome Activation

MeV strains Bilthoven (WT, genotype C2) and Edmonston-Zagreb (EZ, LAMV) were propagated and assayed by plaque formation in Vero cells stably expressing the human MeV receptor SLAMF1 (Vero/hSLAM cells) [38]. Virus stocks were documented free of defective interfering RNA and mycoplasma. Vero/hSLAM cells were cultured in Dulbecco’s modified Eagle medium (DMEM) with 2% FBS, 1% L-glutamine, and 1% penicillin-streptomycin. For infection, cells were washed with PBS before adding virus diluted in RPMI 1640 containing 2% FBS. For control inflammasome induction, cells were treated with the potassium ionophore nigericin (5 μM; MilliporeSigma, Burlington, MA, USA) and TLR4 agonist LPS (1 μg/mL, Cell Signaling) [39]. Media was used as the control. Cells were incubated with virus for 1 h, gently aspirated before adding fresh media and incubation at 37 °C in 5% CO_2_ for 24 h. Culture supernatant fluids were stored at −80 °C for subsequent cytokine, lactate dehydrogenase (LDH) and virus assays. Cells were harvested using non-enzymatic cell dissociation media (CellStripper, Corning, Tewksbury, MA, USA) for 30 min at 37 °C for flow cytometry analysis. 

### 2.3. Flow Cytometry 

Freshly detached macrophages pooled from three wells were placed in 96-well U-bottom plates, washed twice with PBS and stained with Fixable Viability Stain 780 (Cat 565388; BD Bioscience, Franklin Lakes, NJ, USA) on ice for 30 min to separate live/dead cell populations and washed twice with PBS plus 2 mM EDTA. Cells were incubated with Hu Fc Receptor Binding Inhibitor (Cat. 14-9161-73; Thermo Fisher Scientific, Whaltman, MA, USA) on ice for 15 min and then stained with the following antibodies to document macrophage differentiation [37,40]: anti CD14 (M5E2)-BV510, anti CD209 (9E9A8)-BV421, anti CD11c (3.9)-APC, anti CD38 (HIT2)-PE-Cy7, anti-CD11b (ICRF44)-PerCP-Cy 5.5, anti CD206 (15-2)-PE (all from BioLegend, San Diego, CA, USA). Antibodies were diluted in FACS buffer (2% BSA and 2mM EDTA in PBS) and cells stained for 30 min on ice. To detect MeV infection, washed cells were fixed with 4.2% formaldehyde BD Cytofix/ Cytoperm (Cat. 554714, BD Biosciences) for 20 min at 4 °C and stained for the MeV N protein using mouse anti-MeV (83KKII)-FITC (Millipore, Temecula, CA, USA). Data were acquired using a FACSCanto II flow cytometer with FACSDiva Software (BD Biosciences) and analyzed using FlowJo software (Treestar, Ashland, OR, USA). Gating was based on fluorescence minus one. 

### 2.4. Plaque Assay for Infectious Virus 

Supernatant fluids from infected macrophages were serially diluted in DMEM/2% FBS and added to 90% confluent Vero/hSLAM cells in six well plates and incubated at 37 °C with 5% CO_2_ for 1 h before removing the inoculum and overlaying the cells with prewarmed 0.6% bactoagar in MEM with 2% FBS. After incubation for 5 days cells were fixed with 10% formaldehyde, stained with crystal violet and plaques were counted. Amounts of virus are expressed as plaque-forming units (PFU)/mL.

### 2.5. Cytokine and LDH Assays

Cytokines and LDH in supernatant fluids of uninfected and MeV-infected macrophages 24 h after infection were quantified with commercial kits for human IL-18 (MBL, Woburn, MA; limit of detection 12.5 pg/mL), IL-1β (RayBiotech, Peachtree Corners, GA, USA; limit of detection 0.3 pg/mL), TNF-α (ThermoFisher, Waltham, MA, USA; limit of detection 1.7 pg/mL), IL-6 (ThermoFisher; limit of detection 2 pg/mL) and LDH (CyQuant, Thermo Fisher Scientific, Waltham, MA, USA). LDH results are expressed as percent cytotoxicity: (Experimental − Baseline)/(Maximal − Baseline) × 100 with uninfected cells as baseline and lysed cells as maximal LDH release. All kits were used according to manufacturer’s recommendations and measurements were normalized to the average of culture media controls. 

### 2.6. Statistical Analysis

Infections were completed in triplicate. Results were analyzed using GraphPad Prism 8.4.3 software (San Diego, CA, USA). Student’s unpaired *t* test was used to compare WT and vaccine strains of MeV.

### 2.7. Ethical Statement

The buffy coat packs for isolation of primary human monocytes were from a healthy anonymous donor and obtained from the American Red Cross. Researchers and authors had no interaction with the donor and did not know the basic information of the donor. 

## 3. Results

### 3.1. Infection of Differentiated Primary Human Macrophages and THP-1 Cells with WT and Vaccine Strains of MeV 

The molecular basis of attenuation for LAMV is not known. WT and vaccine strains of MeV replicate with similar efficiency in primary endothelial and epithelial cells, but not in cells of the immune system [15,41,42,43]. LAMV infects cultured primary human lymphocytes and monocytes more efficiently than WT MeV, but cells infected at an MOI of 5 produce little virus at 24 h compared to WT MeV-infected cells [15]. To further investigate this restriction and determine whether strain-dependent differences in replication exist for differentiated primary monocyte-derived macrophages and PMA-matured THP-1 cells, we analyzed these cells for WT MeV and LAMV infection using the previous parameters of virus production at 24 h and MOI of 5.

**Figure 1:**
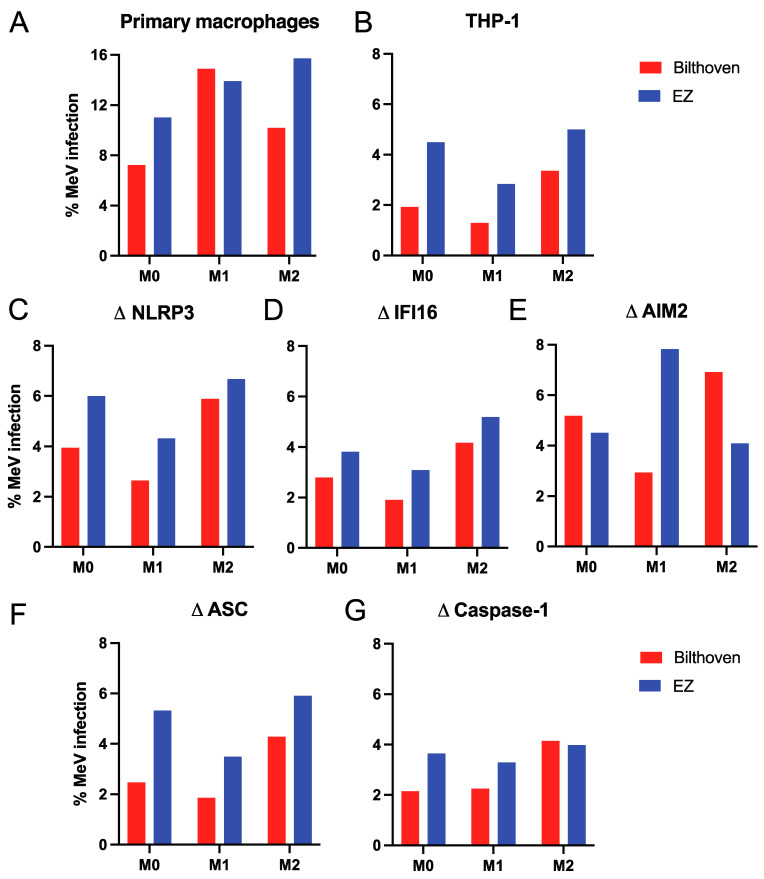
Percentages of macrophages infected with WT and LAMV strains of MeV. Primary monocyte-derived macrophages and PMA-differentiated THP-1 cells (M0) further polarized into inflammatory (M1) macrophages with IFN-γ and LPS or into anti-inflammatory (M2) macrophages with IL-4 were infected with the Bilthoven WT or Edmonston-Zagreb (EZ) vaccine strains of MeV (MOI = 5). Percentages of macrophages positive for MeV N protein 24 h after infection with WT (red) and vaccine (blue) MeV were determined by flow cytometry. (**A**) Primary monocyte-derived macrophages, (**B**) parental THP-1 cells, and (**C**–**G**) THP-1 cells deficient in inflammasome sensors NLRP3 (**C**), IFI16 (**D**) or AIM2 (**E**); adaptor ASC (**F**) or effector caspase 1 (**G**).

Primary human monocytes differentiated into macrophages (M0) with M-CSF and then into M1 macrophages with lipopolysaccharide (LPS) and IFN-γ and into M2 macrophages with IL-4 [37] were infected with WT (Bilthoven) and LAMV (Edmonston-Zagreb, EZ) strains of MeV at an MOI of 5 determined in Vero/hSLAM cells. After 24 h, the percentages of cells infected were determined by flow cytometry analysis of cells stained with antibody to the MeV nucleocapsid (N) protein (Figure 1A). Macrophages were infected either similarly or more efficiently by LAMV than WT MeV independent of differentiation status.

Monocytic THP-1 cells are well characterized and widely used for reproducible analysis of human macrophage infection and inflammasome activation [28,37,44,45]. THP-1 cells were matured into CD11b^+^CD11c^+^ M0 macrophages with PMA which also increases expression of the CD150/SLAMF1 MeV receptor [45]. M0 THP-1 cells were then differentiated into CD38^+^ M1 and CD209^+^ M2 macrophages and infected as above for primary macrophages. EZ vaccine infected higher percentages of THP-1 cells than WT MeV independent of differentiation status (Figure 1B).

To determine whether infection was affected by inflammasome component deficiency THP-1 cells with engineered deletions of sensors NLRP3, IFI16 and AIM2, adaptor ASC or effector caspase 1 [35] were similarly studied (Figure 1C–G) and showed that primary human monocyte-derived macrophages and differentiated THP-1 cells could be infected with both WT and LAMV strains of MeV independent of inflammasome component deficiencies. Overall (81% of the time), macrophages were more likely to be infected by LAMV than by WT MeV independent of differentiation status.

### 3.2. Production of Infectious Virus by Differentiated Primary Human Macrophages and THP-1 Cells Infected by WT and Vaccine Strains of MeV

**Figure 2:**
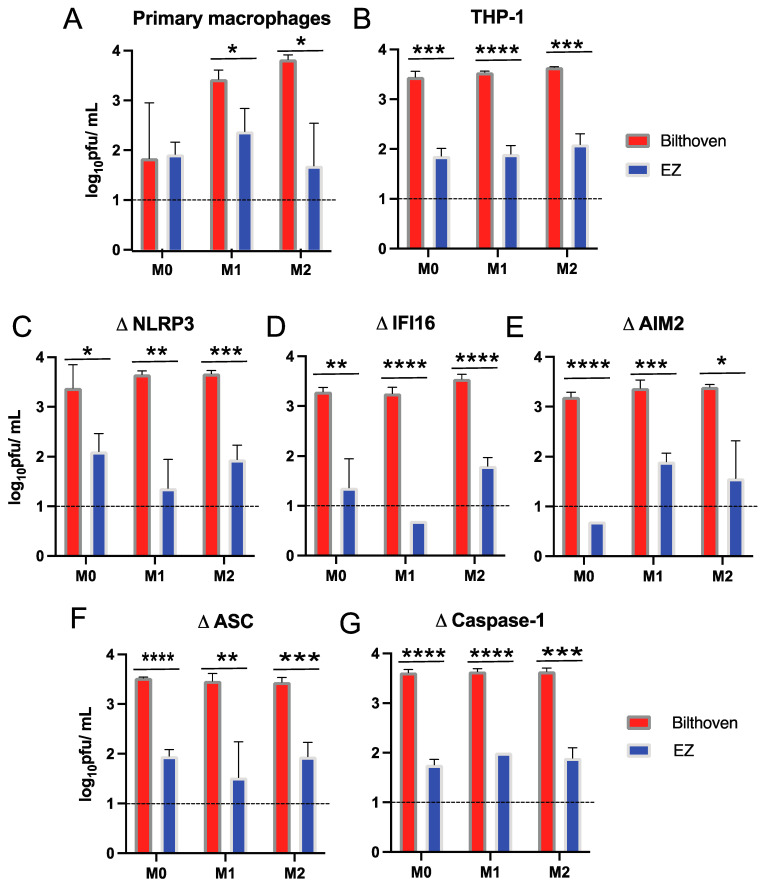
Production of infectious virus by human macrophages infected with WT and vaccine strains of MeV. Primary monocyte-derived macrophages and PMA-differentiated THP-1 cells (M0) further differentiated into inflammatory (M1) macrophages with IFN-γ and LPS or into anti-inflammatory (M2) macrophages with IL-4 were infected with WT (Bilthoven, red) or LAMV (EZ, blue) strains of MeV (MOI = 5). Infectious virus released into culture supernatant fluids 24 h after infection was measured by plaque formation on Vero/hSLAM cells and expressed as plaque-forming units (pfu)/mL. (**A**) Primary monocyte-derived macrophages, (**B**) parental THP-1 cells, and (**C**–**G**) THP-1 cells deficient in inflammasome sensors NLRP3 (**C**), IFI16 (**D**) and AIM2 (**E**); adaptor ASC (**F**) or effector caspase 1 (**G**). Data are presented as geometric means +/− SD of 3 replicate samples. Horizontal line indicates the limit of detection. * *p* < 0.05, ** *p* < 0.01, *** *p* < 0.001, **** *p* < 0.0001, student’s unpaired *t* test.

Culture supernatant fluids collected 24 h after infection of differentiated primary human macrophages and THP-1 cells were assessed for production of infectious virus by plaque assays on Vero/hSLAM cells (Figure 2). Viral progeny production was greater for differentiated M1 and M2 primary macrophages (Figure 2A) and differentiated THP-1 cells (Figure 2B) infected with WT MeV compared to LAMV. This pattern of preferential virus production by WT MeV was not altered by inflammasome component deficiencies (Figure 2C–G). These studies showed that both differentiated primary human monocyte-derived macrophages and THP-1 cells could be infected with WT MeV and LAMV, but efficiently released infectious virus only after WT infection. These traits were independent of functional polarization.

### 3.3. Inflammasome-Induced Release of IL-18 and IL-1β from Macrophages Infected with LAMV and WT Strains of MeV

**Figure 3:**
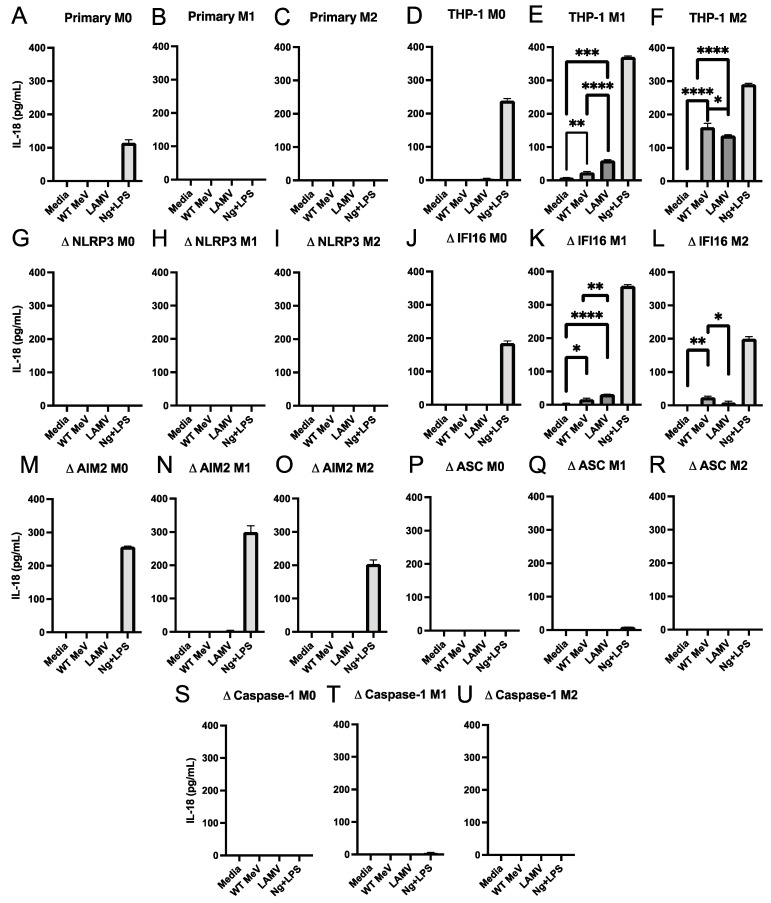
Amounts of IL-18 in culture supernatant fluids from differentiated macrophages infected with WT MeV (Bilthoven) and LAMV (EZ) strains of MeV. Cells were infected (MOI = 5) for 24 h and IL-18 measured by EIA and expressed as pg/mL (limit of detection = 12.5 pg/mL). Primary monocyte-derived macrophages and PMA-differentiated THP-1 cells (M0) were further differentiated into inflammatory (M1) macrophages with IFN-γ and LPS or into anti-inflammatory (M2) macrophages with IL-4. (**A**–**C**) primary monocyte-derived macrophages, (**D**–**F**) parental THP-1 cells, and THP-1 cells deficient in NLRP3 (**G**–**I**), IFI16 (**J**–**L**), AIM2 (**M**–**O**), ASC (**P**–**R**), or caspase-1 (**S**–**U**). Media from uninfected cells served as a negative control and from nigericin (Ng) and LPS-stimulated cells served as a positive control. * *p* < 0.05, ** *p* < 0.01, *** *p* < 0.001, **** *p* < 0.0001, student’s *t* test.

**Figure 4:**
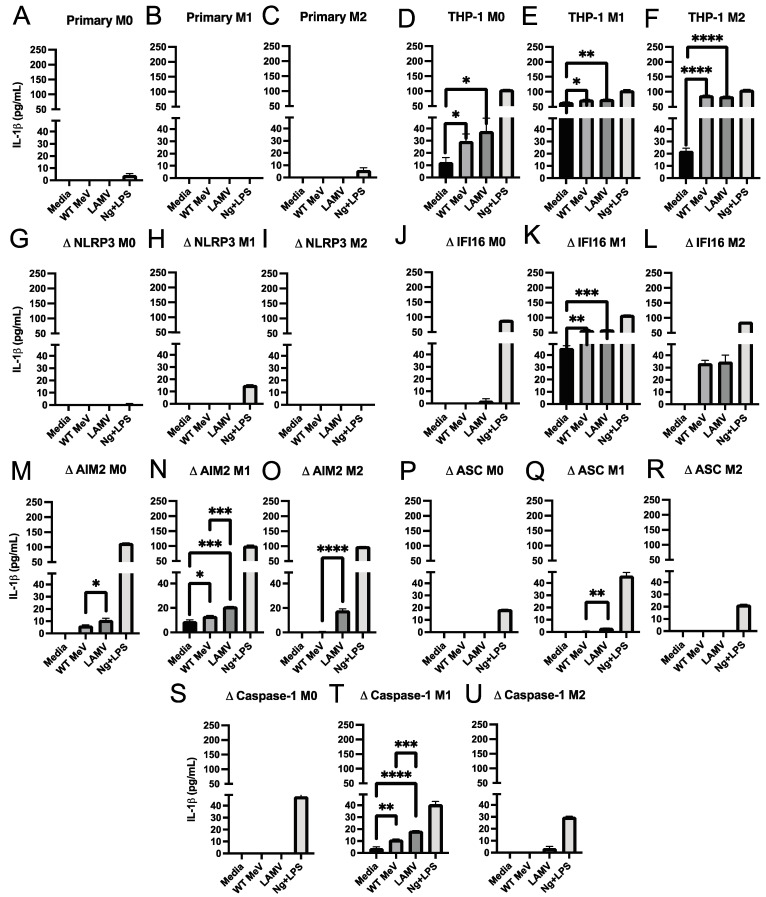
Amounts of IL-1β in culture supernatant fluids from differentiated macrophages infected with WT MeV (Bilthoven) and LAMV (EZ) strains of MeV. Cells were infected (MOI = 5) for 24 h and IL-1β measured by EIA and expressed as pg/mL (limit of detection = 0.3 pg/mL). Primary monocyte-derived macrophages and PMA-differentiated THP-1 cells (M0) were further differentiated into inflammatory (M1) macrophages with IFN-γ and LPS or into anti-inflammatory (M2) macrophages with IL-4. (**A**–**C**) primary monocyte-derived macrophages, (**D**–**F**) parental THP-1 cells, and THP-1 cells deficient in NLRP3 (**G**–**I**), IFI16 (**J**–**L**), AIM2 (**M**–**O**), ASC (**P**–**R**), or caspase-1 (**S**–**U**). Culture media from uninfected cells served as a negative control and from nigericin (Ng) and LPS stimulated cells served as a positive control. * *p* < 0.05, ** *p* < 0.01, *** *p* < 0.001, **** *p* < 0.0001, student’s unpaired *t* test.

To determine whether MeV infection led to inflammasome activation we used enzyme immunoassays (EIAs) to measure the amounts of IL-18 (Figure 3) and IL-1β (Figure 4) produced by WT MeV- and LAMV-infected macrophages with different states of differentiation. Neither IL-18 (Figure 3A–C) nor IL-1β (Figure 4A–C) was detected in culture fluids from WT MeV or LAMV-infected primary monocyte-derived macrophages at 24 h independent of differentiation phenotype but was induced by control treatment with nigericin plus LPS indicating that the cells were able to undergo inflammasome activation. Therefore, MeV-induced activation of inflammasome cytokine processing and release was not detected in primary macrophages within 24 h of infection.

In contrast, IL-18 was detected in culture fluids from LAMV (EZ)-infected M0 (Figure 3D) and WT MeV and LAMV-infected M1 and M2 parental THP-1 cells (Figure 3E,F). Likewise, IL-1β production was also detected after WT MeV and LAMV infection of parental M0, M1 and M2 THP-1 cells (Figure 4D–F) further indicating inflammasome induction by WT MeV and LAMV of both inflammatory and anti-inflammatory THP-1 macrophages.

### 3.4. Effect of Inflammasome Component Deficiencies on MeV-Induced Release of IL-18 and IL-1β from THP-1 Macrophages

To determine the requirements for MeV activation of the inflammasome in THP-1 macrophages, culture supernatant fluids from LAMV and WT MeV-infected THP-1 cells deficient in NLRP3, IFI16, AIM2, ASC, or caspase 1 and differentiated into M0, M1, and M2 phenotypes were assayed for IL-18 (Figure 3) and IL-1β (Figure 4). IL-18 was produced by MeV-infected IFI16-deficient M1 and M2 differentiated THP-1 cells with greater production induced by LAMV in M1 and WT MeV in M2 cells (Figure 3J–L). Only LAMV induced detectable IL-18 production by AIM2-deficient M1 THP-1 cells (Figure 3M–O). No IL-18 was induced by MeV infection of NLRP3, ASC or caspase 1-deficient THP-1 cells (Figure 3G–I,P–U). These results indicated that inflammasome activation by both LAMV and WT MeV leading to the production of IL-18 was dependent on the NLRP3, ASC, and caspase 1 inflammasome and independent of the IFI16 and AIM2 sensors.

Likewise, IL-1β production was detected after WT MeV and LAMV infection of AIM2-deficient (Figure 4M–O) and IFI16-deficient (Figure 4J–L) cells. However, although no detectable IL-1β was produced by WT or vaccine MeV-infected NLRP3-deficient THP-1 cells (Figure 4G–I), M1-differentiated, but not M0 or M2-differentiated THP-1 cells deficient in ASC (Figure 4P–R) produced IL-1β. In addition, M1-, and to a lesser extent M2-differentiated caspase 1-deficient THP-1 cells also produced IL-1β in response to infection (Figure 4S–U). These data indicate that MeV infection can activate ASC and caspase 1-independent pathways for pro-IL-1β-processing in M1 inflammatory THP-1 macrophages.

### 3.5. Cell Death of MeV-Infected Differentiated THP-1 Cells

**Figure 5:**
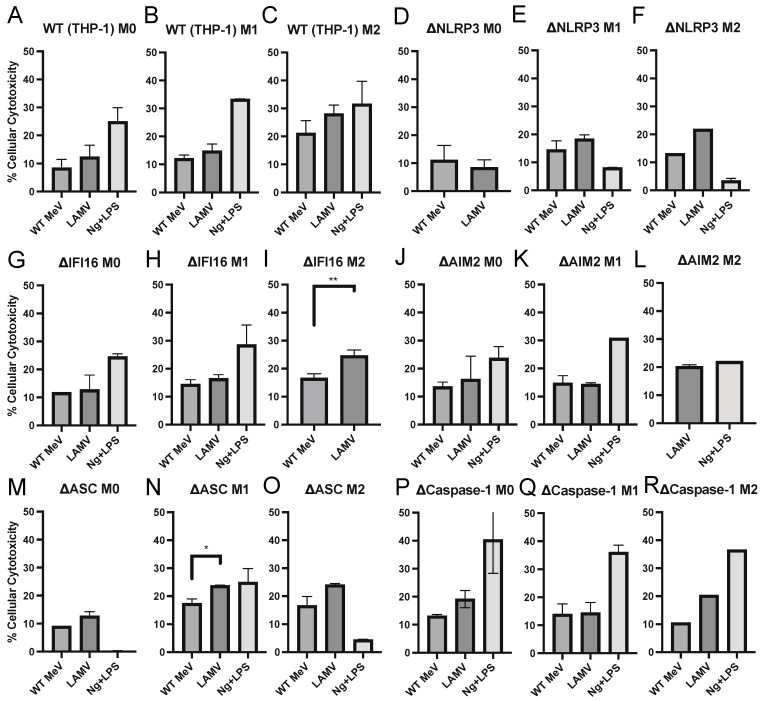
Cytotoxic responses of differentiated macrophages infected with WT (Bilthoven) or LAMV (EZ) strains of MeV or treated with nigericin (Ng) and LPS. PMA-differentiated THP-1 cells (M0) were further differentiated into inflammatory (M1) or into anti-inflammatory (M2) macrophages as previously described. (**A**–**C**) Parental THP-1 cells, and THP-1 cells deficient in NRLP3 (**D**–**F**), IFI16 (**G**–**I**), AIM2 (**J**–**L**), ASC (**M**–**O**), or caspase-1 (**P**–**R**). Cells were infected with viruses (MOI = 5) and at 24 h supernatant fluids were assayed for LDH release. Percentage cellular cytotoxicity was calculated relative to the LDH release by uninfected cells as a baseline and cells treated with a lysis buffer for maximal LDH release. Percent cellular cytotoxicity = (Experimental − Baseline)/(Maximal − Baseline) × 100. Sufficient supernatant fluid was not available for analysis of all samples. * *p* < 0.05, ** *p* < 0.01, student’s unpaired *t* test.

Because inflammasome activation of caspase 1 can lead to pyroptotic cell death [46,47], we assessed the release of LDH from WT MeV and LAMV-infected THP-1 cells 24 h after infection to determine whether MeV activation of the inflammasome resulted in rapid cell death (Figure 5). In general, cell death, as assessed by LDH release, a relatively nonspecific indicator of non-apoptotic cell death, associated with both WT MeV and LAMV infections was limited and unrelated to inflammasome activation although further studies would be required to rule out induction of pyroptosis. The 10–20% calculated cytotoxicity observed after infection of M0, M1 and M2 differentiated THP-1 cells represents an overall assessment of cell health in infected cultures and was observed for cells with and without inflammasome component deficiencies.

### 3.6. Production of IL-6 and TNFα by MeV-Infected Macrophages

**Figure 6:**
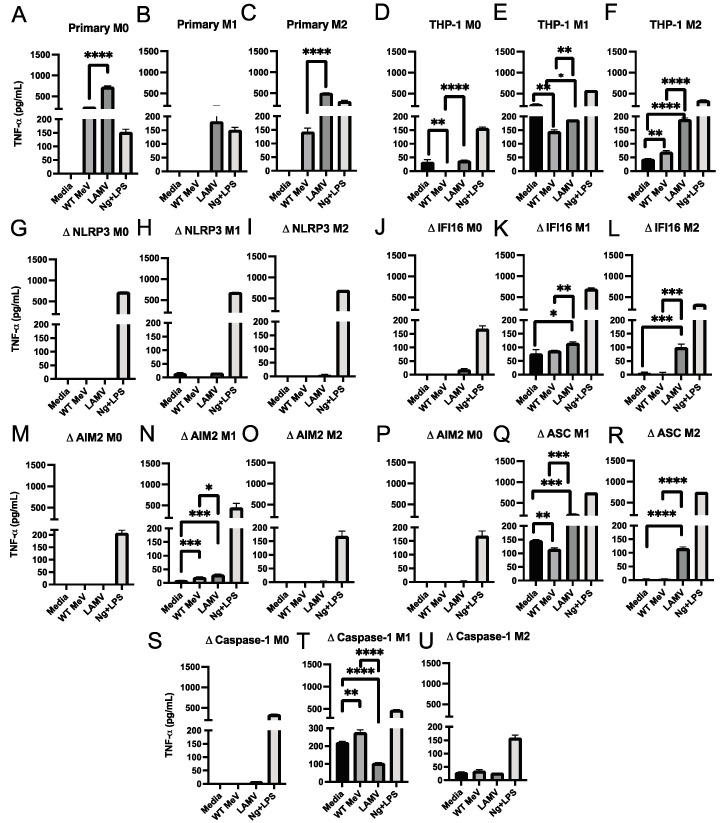
Amounts of TNFα in culture supernatant fluids from differentiated macrophages infected with WT (Bilthoven) and vaccine (EZ) strains of MeV. Cells were infected (MOI = 5) for 24 h and IL-1β measured by EIA and expressed as pg/mL (assay limit of detection = 1.7 pg/mL). Primary monocyte-derived macrophages and PMA-differentiated THP-1 cells (M0) were further differentiated into inflammatory (M1) macrophages with IFN-γ and LPS or anti-inflammatory (M2) macrophages with IL-4. (**A**–**C**) Primary monocyte-derived macrophages; (**D**–**F**) parental THP-1 cells; THP-1 cells deficient in NLRP3 (**G**–**I**), IFI16 (**J**–**L**), AIM2 (**M**–**O**), ASC (**P**–**R**), or caspase-1 (**S**–**U**). Culture media served as a negative control and nigericin (Ng) with LPS as a positive control. * *p* < 0.05, ** *p* < 0.01, *** *p* < 0.001, **** *p* < 0.0001, student’s unpaired *t* test.

**Figure 7:**
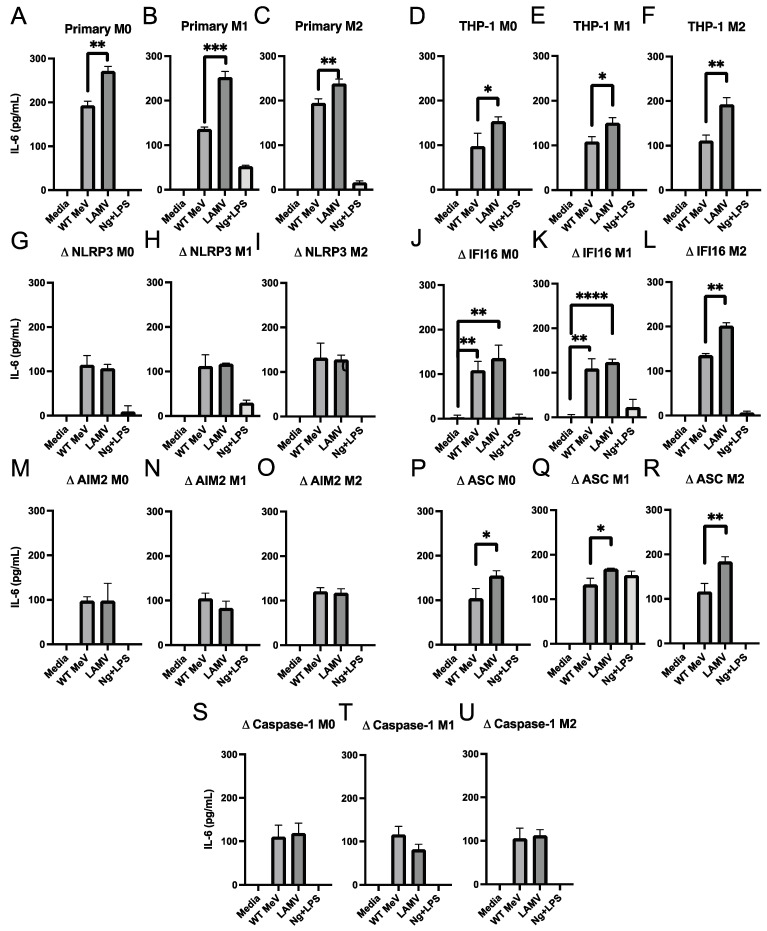
Amounts of IL-6 in culture supernatant fluids from differentiated macrophages infected with WT (Bilthoven) and vaccine (EZ) strains of MeV. Cells were infected (MOI = 5) for 24 h and IL-6 measured by EIA and expressed as pg/mL (assay limit of detection = 2 pg/mL). Primary monocyte-derived macrophages and PMA-differentiated THP-1 cells (M0) were further differentiated into inflammatory (M1) macrophages with IFN-γ and LPS or anti-inflammatory (M2) macrophages with IL-4. (**A**–**C**) Primary monocyte-derived macrophages; (**D**–**F**) parental THP-1 cells; THP-1 cells deficient in NLRP3 (**G**–**I**), IFI16 (**J**–**L**), AIM2 (**M**–**O**), ASC (**P**–**R**), or caspase-1 (**S**–**U**). Culture media served as a negative control. * *p* < 0.05, ** *p* < 0.01, *** *p* < 0.001, **** *p* < 0.0001, student’s unpaired *t* test.

To determine the ability of vaccine and WT strains of MeV to induce macrophage production of NF-κB-dependent inflammasome-independent cytokines characteristic of WT MeV infection in vivo, culture supernatant fluids from M0, M1, and M2 differentiated primary monocyte derived macrophages and THP-1 cells were assayed for TNFα (Figure 6) and IL-6 (Figure 7). Control treatments with nigericin and LPS induced TNFα (Figure 6), but not IL-6 (Figure 7). MeV infection of primary monocyte-derived macrophages (Figure 6A–C and Figure 7A–C) and THP-1 cells (Figure 6D–F and Figure 7D–F) of all differentiation phenotypes induced production of TNFα and IL-6 with higher levels after EZ vaccine than WT MeV infection. Except for NLRP3-deficient THP-1 cells that did not produce detectable TNFα (Figure 6G–I), M1-differentiated THP-1 cells with other inflammasome component deficiencies did produce TNFα, particularly after LAMV infection (Figure 6J–U) and IL-6 similarly after LAMV and WT MeV infection (Figure 7G–U). Therefore, both WT and vaccine strains of MeV induced inflammasome-independent production of TNFα and IL-6 by macrophages in vitro.

## 4. Discussion

To characterize MeV replication and the innate responses of macrophages, a target cell for both WT and vaccine strains of MeV [23,48], we have studied primary human monocyte-derived macrophages and PMA-matured monocytic THP-1 cells polarized to inflammatory (M1) or anti-inflammatory (M2) phenotypes. LAMV infected macrophages more efficiently than WT MeV but LAMV-infected cells produced little virus compared to WT-infected cells independent of polarization state. Both strains induced rapid production of mature IL-1β and IL-18 by THP-1 cells, but not primary macrophages. Analysis of THP-1 cells deficient in inflammasome components showed that MeV-induced IL-18 production was dependent on NLRP3, ASC and caspase 1. In contrast, IL-1β production required NLRP3, but could be produced in the absence of ASC or caspase 1 indicating activation of other pathways for NLRP3-mediated pro-IL-1β processing. Both vaccine and WT strains of MeV induced production of the NF-κB-inducible cytokines IL-6 and TNFα. Thus, an important manifestation of MeV vaccine virus attenuation is restricted production of infectious virus by macrophages. However, NLRP3-dependent in vitro induction of IL-1β and IL-18, as well as NF-κB-dependent IL-6 and TNFα by vaccine and WT MeV is similar and therefore independent of assembly and release of infectious virus.

The mechanism(s) of MeV vaccine virus attenuation is not known, but previous in vivo and in vitro studies of vaccine and WT virus replication have documented restricted replication in lymphoid tissue, rather than altered cell tropism, as the primary manifestation of attenuation [15,48,49,50]. The current studies document a similar phenotype for primary monocyte-derived macrophages and PMA-differentiated THP-1 cells. Limited production of infectious virus is consistent with inefficient replication and spread in lymphoid tissue as the primary in vivo manifestation of vaccine attenuation. The Bilthoven WT and EZ vaccine strains of MeV differ in sequence in most viral proteins with changes common to all vaccine strains in the phospho (P/V/C), matrix (M), hemagglutinin (H), and large polymerase (L) protein genes [15,51,52]. However, no one change or combination of changes has been identified as responsible for attenuation [53,54,55,56,57,58,59]. THP-1 cells may provide an in vitro system for future identification of the determinants of MeV attenuation.

Infection of PMA-matured THP-1 cells by both vaccine and WT MeV was sufficient to trigger inflammasome activation for production of IL-1β and IL-18 independent of extracellular virus production. Inflammasome-mediated production of IL-18 was dependent on NLRP3, ASC, and caspase 1 but not IFI16 or AIM2 consistent with previous studies showing that RNA viruses stimulate the NLRP3 inflammasome while DNA viruses tend to activate inflammasome processing through the AIM2 or IFI16 sensors [60]. Failure of primary human macrophages to produce these cytokines, despite in vivo production, may indicate that another cell is the source or that more prolonged infection is required for full primary cell inflammasome priming and activation. This question requires further investigation.

Previous studies have indicated that inflammasome activation of M0 THP-1 cells for production of IL-1β is dependent on MeV replication and caspase 1 [45]. However, the current studies show that M1-differentiated THP-1 cells can produce IL-1β in response to LAMV infection without production of infectious virus suggesting that the replication step required is prior to virion assembly. Furthermore, IL-1β, but not IL-18, was released from MeV-infected ASC- and caspase 1-deficient M1 THP-1 cells indicating an alternative path for processing pro-IL-1β. Several extracellular and intracellular proteases can process pro-IL-1β to its mature form and may be more available in macrophages with a proinflammatory phenotype. For instance, TLR4-mediated internalization of LPS results in non-canonical inflammasome activation in human monocytes by activation of caspase 4/5 [61,62,63]. Identification of the process used by MeV-infected macrophages for IL-1β processing will require further investigation.

Studies of paramyxovirus inflammasome activation are limited and the mechanism by which MeV activates the inflammasome is not clear. However, the N protein of peste des petits ruminants virus (PPRV), a MeV-related morbillivirus, induces IL-1β secretion by interacting with MyD88 to activate NF-κB signaling and with NLRP3 to trigger inflammasome assembly and activate caspase 1 [64]. In addition, inflammasome assembly can be regulated by paramyxovirus V proteins produced through cell-type dependent editing of the P gene RNA [44,45,65].

Production of inflammatory cytokines TNFα and IL-6 indicative of NF-κB activation and the first step in inflammasome activation was largely independent of the inflammasome although TNFα production was reduced in the absence of NLRP3. Vaccine virus infection induced higher levels of these cytokines than WT infection, but this is not evidenced after in vivo infection where plasma cytokine levels are lower after LAMV infection than WT MeV infection likely due to the less efficient local and systemic spread of virus leading to infection of fewer cells [13,15]. The role of inflammasome activation in the pathogenesis of WT MeV infection is unknown but there is little evidence that virus replication is restricted or cell death induced. However, MeV-induced innate responses likely influence the adaptive responses to infection that lead to virus clearance and protective immunity as IL-18 and IL-1β influence the CD4 and CD8 T cell differentiation phenotypes that characterize recovery from measles [66,67,68,69,70].

## Data Availability

The data that support the findings of this study are available from the corresponding author upon reasonable request.
